# Impact of smoking status on health-related quality of life (HRQoL) in cancer survivors

**DOI:** 10.3389/fonc.2023.1261041

**Published:** 2024-01-04

**Authors:** José Ignacio Nolazco, Bernard A. Rosner, Emily H. Roebuck, Cristiane Decat Bergerot, Elke Rammant, Geetha S. Iyer, Yuzhe Tang, Ra’ad Al-Faouri, Dejan K. Filipas, Michael S. Leapman, Matthew Mossanen, Steven Lee Chang

**Affiliations:** ^1^ Division of Urological Surgery, Brigham and Women’s Hospital, Harvard Medical School, Boston, MA, United States; ^2^ Servicio de Urología, Hospital Universitario Austral, Universidad Austral, Pilar, Argentina; ^3^ Channing Division of Network Medicine, Department of Medicine, Harvard Medical School, Boston, MA, United States; ^4^ Department of Urology, Carolinas Medical Center/Atrium Health, Charlotte, NC, United States; ^5^ Centro de Câncer de Brasília, Instituto Unity de Ensino e Pesquisa, Grupo Oncoclinicas, Brasília, DF, Brazil; ^6^ Department of Human Structure and Repair, Ghent University, Ghent, Belgium; ^7^ Division of Pharmacoepidemiology and Pharmacoeconomics, Department of Medicine, Brigham and Women’s Hospital and Harvard Medical School, Boston, MA, United States; ^8^ Urology Department, Beijing Tsinghua Changgung Hospital, School of Clinical Medicine, Tsinghua University, Beijing, China; ^9^ Department of Surgery, Division of Urology, Beth Israel Deaconess Medical Center, Harvard Medical School, Boston, MA, United States; ^10^ Department of Urology, University Medical Center Hamburg-Eppendorf, Hamburg, Germany; ^11^ Yale School of Medicine, Department of Urology, New Haven, CT, United States; ^12^ Department of Radiation Oncology, Brigham and Women’s Hospital, Boston, MA, United States; ^13^ Lank Center for Genitourinary Oncology, Dana-Farber Cancer Institute, Boston, MA, United States

**Keywords:** smoking, tobacco, health-related quality of life, cancer survivors, behavioral risk factors surveillance system (BRFSS)

## Abstract

**Introduction:**

The Health-Related Quality of Life (HRQoL) often declines among cancer survivors due to many factors. Some cancer patients who smoke before the cancer diagnosis continue this harmful habit, potentially contributing to a more significant decline in their HRQoL. Therefore, this study investigates the association between smoking status and HRQoL in cancer survivors.

**Methods:**

We conducted a cross-sectional study utilizing self-reported cancer history from 39,578 participants of the Behavioral Risk Factor Surveillance System (BRFSS) database, leveraging 2016 and 2020 year questionaries. A multidimensional composite outcome was created to assess HRQoL, integrating four distinct dimensions - general health, mental health, physical health, and activity limitations. After accounting for the complex survey design, logistic regression models were used to analyze the association between smoking status and poor HRQoL, adjusting for demographic, socioeconomic, and health-related confounders.

**Results:**

Our study found that, after adjusting for potential confounders, current smokers exhibited a significantly poorer HRQoL than never smokers (OR 1.65, 95%CI 1.40-1.93). Furthermore, former smokers showed a poorer HRQoL than never smokers; however, this association was not as strong as current smokers (OR 1.22, 95%CI 1.09-1.38).

**Conclusion:**

Our findings highlight the adverse association of smoking with poor HRQoL in cancer survivors, underscoring the importance of healthcare professionals prioritizing smoking cessation and providing tailored interventions to support this goal.

## Introduction

Cancer is a leading cause of death globally, with well-established physical and psychological ramifications for affected individuals (1). The importance of assessing health-related quality of life (HRQoL) in oncological research is gaining recognition. HRQoL is a multidimensional, comprehensive, and complex concept that includes diverse factors that collectively contribute to an individual overall well-being (2, 3). Numerous studies have established a strong association between increased HRQoL and enhanced survival outcomes in cancer patients (4–6). Interestingly, a significant number of patients perceive heightened HRQoL as preferable to an extended survival period (7). However, despite its significance, HRQoL often declines among cancer survivors due to a variety of factors, including physical symptoms like pain, fatigue, nausea, psychological distress, and social isolation (8).

According to a recent study utilizing the National Health Interview Survey (NHIS) dataset, the general population’s smoking prevalence in 2020 was 12.5% (9). In contrast, an investigation encompassing 32,244 cancer survivors from the Population Assessment of Tobacco and Health (PATH) dataset indicated a disconcerting 17.2% smoking prevalence within this cohort (10). It is important to consider that certain malignancies demonstrate a more robust correlation with tobacco consumption relative to others, underscoring the complex relationship between smoking and cancer (11). Smoking persists as a prevalent behavior among cancer survivors and is associated with unfavorable treatment outcomes, including reduced treatment effectiveness, increased risk of recurrence, complications, toxicity, and lower survival rates (12–14).

Nevertheless, there remains a limited understanding of the specific factors influencing health-related quality of life (HRQoL) in cancer survivors, particularly in relation to smoking status. Although prior studies have demonstrated an association between smoking and poor HRQoL in diverse populations (15, 16), few have investigated the association between smoking and HRQoL among cancer patients. Uncovering this relationship is essential to improve the overall HRQoL of these individuals. Consequently, this study aimed to examine the relationship between smoking status and HRQoL in cancer survivors.

## Methods

### Study population

This study utilized data from the Centers for Disease Control and Prevention’s (CDC) Behavioral Risk Factor Surveillance System (BRFSS) for the years 2016-2020, a cross-sectional, state-based telephone survey of non-institutionalized individuals aged 18 years or older residing in the United States (17). The questionnaire contained sections addressing demographics, healthcare access, and health-related behaviors. Cancer survivors were identified through a self-reported history of cancer and those without cancer were excluded from further analyses. The resulting cohort comprised 39,578 adult cancer survivors living in the U.S. with at least one self-reported HRQoL proxy (general health, mental health, physical health, and activity limitations). The included cancers were brain, bladder, bone, breast, colon, cervical, endometrial, esophageal, gastric, Hodgkin’s lymphoma, leukemia, liver, lung, melanoma, non-Hodgkin’s lymphoma, oral, ovarian, pharyngeal, pancreatic, prostate, rectal, renal, testicular, thyroid, and other skin cancers. Due to the small sample size (fewer than 100 cases), laryngeal, heart, and neuroblastomas were excluded from the study.

### Exposure variable

The exposure variable was defined as smoking status. Exposure to smoking status was defined into three distinct categories: never smokers, former smokers, and current smokers, based on participants’ responses to two survey questions. (a) “Have you smoked at least 100 cigarettes in your entire life?” Respondents who answered “no” were classified as never smokers. Those who answered “yes” to this question were further divided based on their response to a second question: (b) “Do you now smoke cigarettes every day, some days, or not at all?” Participants who replied “not at all” were classified as former smokers, while those who answered “every day or some days” were designated as current smokers.

### Outcome and variables

The primary composite outcome measure was the HRQoL. The participants’ self-reported HRQoL was assessed using the core section of the survey, which included questions on four domains: general health, mental health, physical health, and activity limitations (**Supplementary Table 1**). These validated questions have previously been used to provide reliable HRQoL estimates (18). The self-assessed general health status was dichotomized into “fair/poor” and “excellent/very good/good.” The other three HRQoL variables were dichotomized based on their frequency of occurrence in the preceding 30 days, with those reporting fewer than 14 (good) and 14 days or more (poor) following the approach used in earlier studies on this topic (19, 20).

The composite outcome was created by first evaluating the validity and reliability of the measurement instrument using Cronbach’s alpha coefficient (21). An alpha score > 0.6 was considered indicative of a valid instrument for measuring HRQoL (22). The resulting Cronbach’s alpha was 0.658, suggesting that the composite outcome was appropriate for the HRQoL assessment. To create the composite outcome “HRQoL,” we computed the row mean of the four dichotomized domains. In assessing HRQoL, participants were partitioned into two distinct groups based on their HRQoL scores. Individuals with an HRQoL score below 0.5 were assigned to the “poor HRQoL” category (HRQoL < 0.5), while those who scored 0.5 or higher were assigned to the “good HRQoL” category (HRQoL ≥ 0.5). This cut-off value was chosen to better identify patients with poorer quality of life, following the approach of dichotomizing the composite outcome into better and poorer halves, as employed by other researchers (23).

The explanatory variables included smoking status, demographic factors (age, gender, race/ethnicity, and marital status), socioeconomic factors (healthcare insurance, employment status, education level, and income), and comorbidities [body mass index (BMI), cardiovascular disease (myocardial infarction, stroke, or coronary heart disease), diabetes, asthma, and type of cancer]. All of these variables were considered during the analysis to assess HRQoL outcomes among cancer survivors.

### Statistical analysis

Descriptive statistics, including frequencies and percentages, were used to present the categorical variables, and chi-square tests were employed to examine the differences between the two groups by evaluating the distribution of these variables. A complex survey design was considered by adjusting for stratification and clustering at the primary sampling unit, using sampling weights to compute nationwide representative frequencies and proportions. Multiple imputations were conducted using the predictive mean-matching method to address missing values, with k = 5 imputations.

A logistic regression model was used to calculate the odds of having poor HRQoL among cancer survivors based on their smoking status (never, former, and current smokers), adjusting for multiple potential confounders based on the aforementioned covariates. The predictive probability of poor HRQoL for each smoking exposure group was calculated. Secondary analyses explored the effects of tobacco-related cancers (TRC) and non-TRC on HRQoL, as well as potential interactions between HRQoL, age, and gender. Statistical significance was determined at α < 0.05, with the data analyzed using STATA/BE version 17.0.

### Sensitivity analysis

Several sensitivity analyses were performed to ensure robustness of the findings. First, an analysis excluding missing data was conducted to evaluate the potential influence of incomplete information on the results. Subsequently, two alternative HRQoL dichotomizations were examined. The first dichotomization classified participants as having “good health” if they scored 1 in all self-reported dimensions, while those with a score lower than 1 were considered “poor HRQoL.” The second dichotomization categorized participants with an HRQoL score of 0 as having “poor HRQoL” (**Table 1**).

**Table 1:** Multivariate analysis of the association between smoking status and health-related quality of life at different cut-off points.

	HRQoL = 0/HRQoL > 0			HRQoL <0.5/HRQoL ≥ 0.5			HRQoL < 1/HRqoL = 1		
	OR	*CI*	p-value	OR	*CI*	p-value	OR	*CI*	p-value
Smoking Status									
Never Smoker	*Ref*			*Ref*			*Ref*		
Former Smoker	**1.22**	1.07 -1.39	< 0.001	**1.22**	1.09 -1.38	< 0.001	**1.22**	1.10 - 1.33	< 0.001
Current Smoker	**1.65**	1.39 -1.96	< 0.001	**1.65**	1.40 -1.92	< 0.001	**1.73**	1.51 - 1.98	< 0.001

Multivariate analysis of the association between smoking status and health-related quality of life (HRQoL) at different cut-off points. HRQoL cut-offs: HRQoL = 0 or HRQoL > 0, HRQoL < 0.5 or HRQoL ≥ 0.5, HRQoL < 1 or HRQoL = 1.

CI, confidence interval.

OR, odds ratio.

## Results

### Study population

The sample consisted of 2,193,981 participants surveyed between 2016 and 2020, of whom 39,578 were cancer survivors. The weight of this sample was estimated to represent 13,836,840 cancer survivors. Regarding the exposure status, 9.76% were current smokers, 36.64% were former smokers, and 53.60% had never smoked. **Table 2** shows the differences between groups according to their demographic and health-related characteristics. Compared to never smokers, current smokers were, on average, younger, more likely to be single, have a lower education level, and have a lower income. A significant racial disparity in smoking status among cancer survivors was observed. Specifically, a greater proportion of White survivors were never or former smokers, compared to higher rates of current smoking observed in Black, Hispanic, and other racial groups. Furthermore, current smokers had a higher prevalence of comorbidities such as cardiovascular disease (CVD), diabetes, and asthma.

**Table 2:** Baseline Characteristics of Cancer Survivors by Smoking Status (n=39,578).

Cancer Survivors Characteristics	Never Smokers (n=20,733) No. Column %		Former Smokers (n=14,756) No. Column %		Current Smokers (n=4,089) No. Column %		p-value
**Age**							**<0.001**
< 40	497	2.4	225	1.5	324	7.9	
40 - 49	982	4.7	464	3.1	406	9.9	
50 -59	2904	14.0	1423	9.6	865	21.2	
60 -69	5958	28.7	3909	26.5	1385	33.9	
70 -79	6324	30.5	5538	37.5	907	22.2	
≥ 80	4068	19.6	3197	21.7	202	4.9	
**Gender**							**<0.001**
Female	13276	64.0	7491	50.8	2558	62.6	
Male	7454	36.0	7262	49.2	1530	37.4	
**Race**							**<0.001**
White	18616	89.8	13249	89.8	3384	82.8	
Black	778	3.8	518	3.5	237	5.8	
Hispanic	257	1.2	209	1.4	125	3.1	
Other*†*	1082	5.2	780	5.3	343	8.4	
**Marital Status**							**<0.001**
Single	8063	38.9	6242	42.3	2322	56.8	
Married or Partner	12669	61.1	8514	57.7	1767	43.2	
**Education Level**							**<0.001**
< High School Diploma	659	3.2	816	5.5	434	10.6	
High School Diploma	10085	48.7	8451	57.4	2866	70.2	
College Graduate	9963	48.1	5461	37.1	781	19.1	
**Employment status**							**<0.001**
Yes	7860	38.1	4074	27.7	1474	36.2	
No	12793	61.9	10633	72.3	2603	63.8	
**Income (USD)**							**<0.001**
< 25.000	3105	18.3	2667	21.5	1522	42.8	
25.000 - 50.000	4325	25.5	3658	29.5	995	28.0	
> 50.000	9527	56.2	6084	49.0	1037	29.2	
**Insurance**							**<0.001**
Yes	20284	98.0	14469	98.2	3788	92.9	
No	410	2.0	268	1.8	291	7.1	
**Body mass index**							**<0.001**
Underweight (< 18.5)	274	1.3	178	1.2	175	4.3	
Normal (≥ 18.5 < 25)	6126	29.5	4001	27.1	1433	35.0	
Overweight (≥ 25 < 30)	7346	35.4	5426	36.8	1276	31.2	
Obese (> 30)	6987	33.7	5151	34.9	1205	29.5	
**CVD** *‡*							**<0.001**
Yes	114	0.6	188	1.3	70	1.8	
No	20310	99.4	14272	98.7	3917	98.2	
**Diabetes**							**<0.001**
Yes	3569	17.7	2975	20.8	707	17.9	
No	16613	82.3	11359	79.2	3252	82.1	
**Asthma**							**<0.001**
Yes	2806	13.6	2091	14.2	796	19.6	
No	17883	86.4	12628	85.8	3275	80.4	

HRQoL, Health-Related Quality of Life.

† Consists of Asian, Alaskan, and Native Americans.

‡ Self-reported cardiovascular disease (CVD), including (myocardial infarction, stroke, or coronary heart disease).

Additional details regarding participant characteristics are presented in **Table 2**. Multiple imputations were used to address missing values, representing 16.80% for the income variable, 2.80% for diabetes, 1.80% for CVD, and less than 0.40% for employment status, asthma, insurance, education, gender, and marital status.

### Smoking status and HRQoL

**Figure 1:**
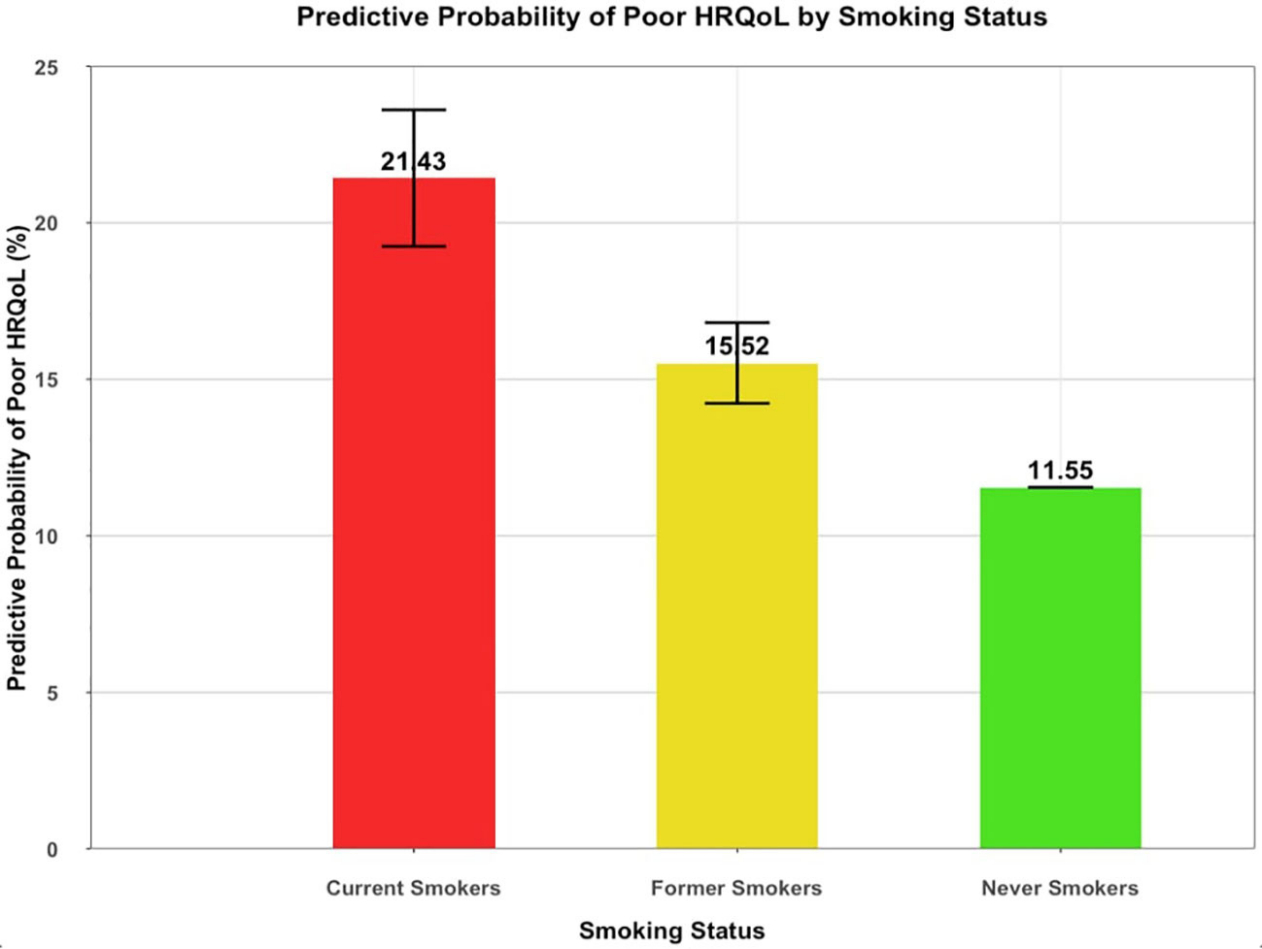
Predictive Probability of Poor HRQoL by Smoking Status Among Cancer Survivors. (n=39,578). Predictive probability of Poor HRQoL among cancer survivors according to smoking status after adjusting for covariates (age, gender, race/ethnicity, marital status, healthcare insurance, employment status, education level, income, and comorbidities [body mass index (BMI) cardiovascular disease (myocardial infarction, stroke or coronary heart disease), diabetes, asthma, and type of cancer). HRQoL encompasses General Health, Mental Health, Physical Health, and Activity Limitations.

In our multivariate analysis, we found that smoking status was an independent predictor of HRQoL in cancer survivors. Our results indicated that being a current or former smoker was significantly associated with reduced HRQoL compared to never smokers. Furthermore, the relationship between smoking and HRQoL was even stronger among current smokers, who had 65% higher odds of having a poor HRQoL than never-smokers OR of 1.65 (95% CI 1.40-1.93). Moreover, former smokers also exhibited a higher probability of poor HRQoL compared to never smokers, with an OR of 1.22 (95% CI 1.09-1.38). (**Table 3**) The predictive probabilities of poor HRQoL were 11,55%, 15.52%, and 21.43% for never, former, and current smokers, respectively (**Figure 1**).

**Table 3:** Association between Smoking Status and Poor HRQoL: Univariate and Multivariate Analysis.

	Univariate			Multivariate*		
Smoking Status	OR	*CI*	p-value	OR	*CI*	p-value
Never Smoker	*Ref*			*Ref*		
Former Smoker	**1.43**	1.27 -1.60	< 0.001	**1.22**	1.09 -1.38	< 0.001
Current Smoker	**2.54**	2.19 - 2.94	< 0.001	**1.65**	1.40 -1.93	< 0.001

*Multivariate analysis adjusted by: age, gender, race/ethnicity, marital status, healthcare insurance, employment status, education level, income, and comorbidities [body mass index (BMI) cardiovascular disease (myocardial infarction, stroke or coronary heart disease), diabetes, asthma, and type of cancer].

CI = confidence interval.

OR = odds ratio.

Poor HRQoL was defined as a composite outcome of HRQoL with a score below 0.5, based on a validated measurement instrument using self-assessed general health status, mental health, physical health, and activity limitation domains.

Impact of type of cancer: tobacco-related cancers (TRC) vs. non-tobacco-related cancers (non-TRC) on HRQoL.

We investigated the relationship between HRQoL and the TRC and non-TRC groups. (**Supplementary Table 2**) We found that patients with a TRC had significantly greater odds of having a poor HRQoL than those with non-TRC, with an OR of 1.51 (95%CI:1.32-1.72; p < 0.001).

### Interactions

We evaluated the interactions between smoking status and tobacco-related cancers in predicting the HRQoL. The results indicated no significant interaction between the two variables. Additionally, we examined potential interactions between gender and age; however, none were significant predictors of HRQoL.

### Sensitivity analysis

In the sensitivity analysis, we excluded all missing data and discovered that the outcomes were consistent with the initial analysis after multiple imputations. The odds of poor HRQoL were 80% higher for current smokers than never smokers, with an OR of 1.80 (95% CI 1.46-2.22). Former smokers exhibited a tendency toward lower HRQoL compared to never smokers, with an OR of 1.14 (95% CI 0.98-1.33), although this relationship was not statistically significant (p = 0.082). In our second sensitivity analysis, we dichotomized HRQoL into two alternative categories. The first one defined good HRQoL as those participants with a score of 1 (representing those who had self-reported good health across all self-reported dimensions) and participants with a score lower than 1 (representing those with at least one of these dimensions affected). This first alternate dichotomization was consistent with previous findings that former and current smokers had higher odds of poorer HRQoL than non-smokers, as evidenced by the ORs being 1.21 (95% CI:1.10 -1.33) for former smokers and 1.73 (95% CI:1.51 -1.98) for current smokers. We also explored an alternative classification, segregating participants with a score of 0, which typified the poorest HRQoL, from those with a score higher than 0. Comparable results emerged, as smoking was associated with a decline in HRQoL. This connection was evidenced by an OR of 1.22 (95% CI 1.07-1.39) for former smokers and an OR of 1.65 (95% CI 1.39-1.96) for current smokers (p < 0.001) (**Table 1**).

## Discussion

This study found that smoking is strongly associated with poor HRQoL among cancer survivors. The prevalence of current smoking was approximately 10%, and that of former smokers was 37%, indicating that one-fourth of cancer survivors were currently smoking after cancer diagnosis. The results demonstrated that smoking status is an independent predictor of HRQoL in cancer survivors. After adjusting for demographic, socioeconomic, and health-related aspects, our study determined that current smokers had a 65% heightened risk of poor HRQoL, whereas former smokers had a 22% increased likelihood of poor HRQoL compared to never smokers within the cancer survivor population.

Considering that HRQoL is an essential element in cancer care and has a strong association with survival rates and treatment results (24–27), discerning the factors affecting HRQoL among cancer survivors is indispensable for enhancing their overall welfare and sustained health (28, 29). Previous studies established an association between smoking and reduced HRQoL in the general population and patients with diverse medical conditions (30–32). (33, 34) However, few studies have addressed the impact of smoking on cancer survivors’ HRQoL. Our study adds to the literature by specifically examining the effect of smoking on HRQoL in a large sample representative of the U.S. cancer survivor population.

The interplay between smoking and Health-Related Quality of Life (HRQoL) among oncology patients warrants meticulous investigation to elucidate the complex pathways through which tobacco consumption exerts deleterious effects on individual well-being. One potential reason for the harmful impact of smoking on HRQoL is its connection to other unhealthy habits like not being physically active, having poor sleep habits, consuming excessive alcohol, and making suboptimal dietary choices. These modifiable risk factors have consistently demonstrated associations with heightened morbidity and mortality rates (35). Moreover, there is accumulating evidence to suggest that tobacco attenuates the efficacy and tolerability of cancer therapies, potentially *via* mechanisms involving oxidative stress and modulation of drug-metabolizing enzymes, thus leading to a higher risk of cancer recurrence and progression (36, 37). Concomitantly, the burden of comorbidities attributable to smoking can profoundly influence the aggregate morbidity and mortality experienced by this patient population.

Our study also found that current smokers had a significantly higher likelihood of experiencing poor HRQoL than never smokers, with a predictive probability of 21.43% versus 11.55%, respectively. Moreover, former smokers had poorer HRQoL than those who never smoked but were not as bad as current smokers. These results highlight the significance of providing smoking cessation education to cancer survivors and emphasize that quitting is never too late. Given the increased risk of cancer progression, recurrence, second primary malignancies, and inferior treatment outcomes, smoking cessation should be a top priority in managing cancer patients who smoke (38, 39).

While several factors like comorbidities, education, and income level are non-modifiable, smoking is a modifiable risk factor that offers a tangible area for supportive care interventions (40). Therefore, healthcare professionals must prioritize smoking cessation counseling for all cancer patients regardless of whether their cancer is tobacco-related or not, based on the significant potential impact of quitting smoking on HRQoL, cancer outcomes, and overall health in cancer patients (41). Notably, tailored smoking cessation interventions are recommended for cancer patients as an integral component of their cancer care by multiple organizations, such as the National Comprehensive Cancer Network (NCCN), American Society of Clinical Oncology (ASCO), US Preventive Services Task Force (USPSTF), and Centers for Disease Control and Prevention (CDC) (42–44). (45) These institutions advocate that healthcare professionals evaluate tobacco use among all cancer patients and administer evidence-based strategies, including pharmacotherapy and behavioral counseling, to enhance overall health outcomes and quality of life.

A thorough understanding of the association between smoking and its impact on HRQoL in cancer survivors, and an evaluation of the socioeconomic burden associated with smoking-related health costs and loss of productivity can provide a comprehensive understanding of the detrimental effects of smoking on society and the healthcare system. This valuable insight can be utilized to create effective strategies and health policies to lessen this considerable burden, consequently improving HRQoL for cancer survivors and potentially abating the economic strain on the healthcare system (46, 47).

Our study has notable strengths, including its large sample size of 39,578 cancer survivors and its use of the world’s largest continuously conducted health survey by the CDC (48). Additionally, we employed a composite outcome to analyze the multidimensional concept of HRQoL and conducted a Cronbach’s test to ensure instrument measure validity and reliability. However, as with any other study our study has some limitations. First, the cross-sectional design precluded the ability to establish a causal relationship between smoking status and HRQoL. It is necessary to conduct longitudinal investigations to obtain a more profound comprehension of this association. Second, our reliance on self-reported data was subject to potential misclassification due to participants’ memory recall. Third, we must recognize that while our logistic regression model accounts for numerous demographic, socioeconomic, and health-related aspects, HRQoL remains a nuanced and multifarious notion. In this context, additional unmeasured confounders may influence the outcome. Fourth, our investigation is susceptible to right censoring, as excluding the most severe cancer cases, possibly attributable to mortality, may introduce a bias to the findings. Fifth, it is essential to recognize the restricted generalizability of our findings, given that our investigation concentrated on a cohort of cancer survivors residing in the United States. Consequently, the outcomes may not be seamlessly applicabble to cancer survivor populations in other countries. Sixth, our research did not consider temporality, thus rendering it impossible to determine whether cancer survivors had ceased smoking before or after their diagnosis.

In this age of precision medicine, the imperative need to integrate patient-reported outcomes, socio-environmental determinants of health, life quality assessments, nutritional considerations, and behavioral data into oncological research is increasingly evident. With a multitude of diverse and competing treatment strategies available, it is imperative to tailor indications to reflect the personalized needs of patients. Integrating these non-clinical data into the treatment decision-making process is crucial for achieving this objective. In light of this, healthcare providers must diligently evaluate and track HRQoL at an early stage and longitudinally. Further research should encompass a broader spectrum of HRQoL factors, including pain and social and emotional support, to gain deeper insights into their influence on treatment outcomes. Other investigators have sought to understand and address the HRQoL of patients in clinical practice. For example, the National Comprehensive Cancer Network (NCCN) advises incorporating distress management and HRQOL interventions into routine practice. This suggests using the “Distress Thermometer tool” to screen for distress in every medical encounter. This instrument evaluates various domains, including physical symptoms, emotional well-being, family or interpersonal issues, spiritual concerns, financial distress, and functional limitations (49, 50).

In conclusion, our study showed a robust association between smoking status and a negative impact on cancer survivors’ HRQoL. The practical implications of our findings cannot be understated, as it calls for prompt interventions to help cancer survivors quit smoking and improve their HRQoL. As such, healthcare providers must acknowledge the detrimental effects of smoking on HRQoL and take proactive steps to facilitate smoking cessation in this population. Nonetheless, the intricate relationship between smoking status and HRQoL among cancer survivors warrants further investigation, and the onus remains on the research community to unravel this intricate association.

## Data availability statement

Publicly available datasets were analyzed in this study. This data can be found here: https://www.cdc.gov/brfss/annual_data/annual_data.htm.

## Author contributions

JN: Conceptualization, Data curation, Investigation, Methodology, Project administration, Validation, Visualization, Writing – original draft. BR: Conceptualization, Data curation, Formal Analysis, Methodology, Supervision, Writing – review & editing. ER: Visualization, Writing – original draft, Writing – review & editing. CB: Visualization, Writing – original draft, Writing – review & editing. ER: Visualization, Writing – original draft, Writing – review & editing. GI: Investigation, Methodology, Writing – review & editing. YT: Conceptualization, Data curation, Formal Analysis, Software, Writing – original draft. RA: Software, Writing – review & editing. DF: Data curation, Investigation, Software, Visualization, Writing – original draft, Writing – review & editing. ML: Supervision, Validation, Visualization, Writing – review & editing. MM: Investigation, Supervision, Validation, Writing – review & editing. SC: Conceptualization, Formal Analysis, Investigation, Methodology, Resources, Supervision, Validation, Visualization, Writing – original draft.
