# Supplementary material for: Impact of smoking status on health-related quality of life (HRQoL) in cancer survivors
Source: Front Oncol. 2024 Jan 4;13:1261041. doi: 10.3389/fonc.2023.1261041 (PMC10795065; doi:10.3389/fonc.2023.1261041)
Supplement: Supplementary file 1 [file Table_1.docx]

Supplementary Material

# Supplementary Data

***Supplementary Table 1.*** Health-Related Quality of Life (HRQoL) survey questions in the BRFSS.

| **HRQoL Outcome** | **BRFSS Question** |
| --- | --- |
| **General Health** | “Would you say that in general your health is: excellent, very good, good, fair, poor?” |
| **Mental Health** | “Now thinking about your mental health, which includes stress, depression, and problems with emotions, for how many days during the past 30 days was your mental health not good?” |
| **Physical Health** | “Now thinking about your physical health, which includes physical illness and injury, for how many days during the past 30 days was your physical health not good?” |
| **Activity Limitation** | “During the past 30 days, for about how many days did poor physical or mental health keep you from doing your usual activities, such as selfcare, work, or recreation?” |

***Supplementary Table 2.*** *Tobacco-Related Cancers and Non-Tobacco-Related-Cancers*

| **Tobacco-Related Cancers***†* | **Non-Tobacco Related-Cancers** |
| --- | --- |
| Lung | Breast |
| Laryngeal* | Endometrial |
| Mouth | Ovarian |
| Esophagus | Thyroid |
| Throat | Hodgkin Lymphoma |
| Bladder | Non-Hodking Lymphoma |
| Kidney | Prostate |
| Liver | Testicular |
| Stomach | Melanoma |
| Pancreas | Other skin Cancer |
| Colon | Bone |
| Rectum | Brain |
| Cervix |  |
| Acute Myeloid Leukemia |  |

*Note.*

*† TRC according to the National Cancer Institute (NIH)* Adapted from the Nation Cancer Institute (NIH) website. <https://www.cancer.gov/about-cancer/causes-prevention/risk/tobacco#:~:text=Tobacco%20use%20causes%20many%20types,well%20as%20acute%20myeloid%20leukemia>

*Laryngeal cancer (heart and neuroblastoma) were excluded due to the small sample size.
